# Association of integration with oral health among Indian migrants living in the Netherlands

**DOI:** 10.1371/journal.pone.0298768

**Published:** 2024-03-07

**Authors:** Amandeep Pabbla, Charles Agyemang, Geert van der Heijden, Denise Duijster

**Affiliations:** 1 Department of Oral Public Health, Academic Centre for Dentistry Amsterdam (ACTA), University of Amsterdam and VU University, Amsterdam, The Netherlands; 2 Department of Public Health, Academic Medical Centre (AMC), University of Amsterdam, Amsterdam, The Netherlands; 3 Division of Endocrinology, Diabetes, and Metabolism, Department of Medicine, Johns Hopkins University, Baltimore, Maryland, United States of America; Hamadan University of Medical Sciences School of Dentistry, ISLAMIC REPUBLIC OF IRAN

## Abstract

**Background:**

Limited data exist about the relationship between acculturation and oral health. Hence, the aim of this study was to assess the association of integration with self-reported oral health, behaviours, and oral healthcare utilization among Indian migrants living in the Netherlands, a cross sectional survey study.

**Methods:**

Between February and April 2021, a random sample from Dutch municipalities was obtained for the Indian migrants living in the Netherlands (n = 147). A validated questionnaire was used to collect information on independent variables, namely socio-demographic, integration assessment tool: Immigration Policy Lab (IPL-12) and everyday discrimination scale (EDS). The outcome variables were self-reported oral health, oral health behaviours, and oral healthcare utilization. Multiple regression analysis was used to assess the associations.

**Results:**

Higher integration among Indian migrants was associated with longer stay in the Netherlands, having a Dutch passport, intention to settle in the Netherlands, and having a permanent residence. After adjusting for covariates such as age, gender, marital status, education, income, occupation, and dental insurance, regression analysis showed that Indians with higher integration had lower odds of reporting their oral health as fair to poor [OR = 0.92(95%CI:0.0.85;0.99)] than the Indians with low integration scores. Also, Indians with higher integration had lower odds of using a manual toothbrush as compared to an electric toothbrush or use of both [OR = 0.86(95%CI:0.76;0.97)]. Highly integrated Indians had lower odds of consuming Indian sweets than lower integrated Indians (OR = 0.91; 95%CI:0.86;0.97). Indians with higher integration had 1.15 times (95% CI:1.03;1.29) higher odds of visiting a Dutch dental professional than visiting a dentist in both places (India and the Netherlands). No significant association was found between discrimination and the three outcome variables.

**Conclusion:**

Integration is positively association with self-reported oral health outcomes among the Indian migrants. Measure to improve integration among Indian migrants may help to promote healthy oral health behaviours and improve their oral health care utilization.

## Introduction

According to the report by Global Commission on International Migration (GCIM), almost one in every 10 persons living in high-income countries is a migrant. Europe alone is home to 82 million international migrants, many of whom are from Asian or African backgrounds [1]. Irrespective of the type of migration or reason to migrate, this mobility of people leads to interactions between diverse cultures, which generally results in sharing of beliefs, social norms, values, and traditional practices between distinct groups [2]. This concept has been referred to as acculturation, a term borrowed from anthropology and defined as changes in beliefs, values, identity, or behaviours such as language, customs, diet, or social relationships that occur in minority-culture individuals (migrant or indigenous) as a result of prolonged contact with the majority culture [3]. From the migrant’s perspective, culturally being exposed to the host society, has the ability to change their personal beliefs, values, and practices [4]. These complex interactions have been put forward as acculturation strategies by Berry et al. [5] as assimilation (movement toward the dominant culture), rejection (reaffirmation of the traditional culture), integration (synthesis of the two cultures), or marginalization (alienation from both cultures).

Since the impact of acculturation can be observed in all domains of migrant life, its role in the health of the migrants cannot be overlooked. In fact, research on this association started in the 1960’s and since then the impact of acculturation on migrant health has been widely documented across the globe [6–9]. In contrast, research related to impact of acculturation on oral health gained momentum only during the last two decades. Dahlan et al. [10]. observed a positive association between acculturation and oral health behaviours, wherein proxy measures of acculturation such as language spoken by migrants and length of stay at the host country were used. Another systematic review also reported positive associations between at least one acculturation indicator, namely country of birth, age at immigration, language proficiency, duration, and reason for immigration, with higher use of dental services among migrants [11].

However, use of the abovementioned proxy measures of acculturation alone, makes it difficult to assess the extent (level and type) of acculturation among the migrants [9, 10]. One possible outcome of acculturation as defined by Berry is integration: synthesis of the two cultures [5] This outcome has been shown to be a more reliable and rational measure of how well migrants adapt to the host country [4, 12]. A scale developed by Harder et al, called the Immigration Policy Lab (IPL) integration index, specifically measures the level of integration among migrant groups [13]. This index is comprehensive in its scope, covering psychological, economic, linguistic, navigational, political, and social domains that are integral to the migrant’s integration.

Also, the definition of a migrant group is unclear in literature. This results in discrepant findings in oral health outcomes [14]. It has been recommended to focus on migrant groups that share some cultural and ethnic characteristics with each other. The Netherlands is increasingly becoming a destination of choice for knowledge and family migrants, especially from countries like India. As of 2019, an estimated 58,460 Indians (exclusive of Surinamese Hindustanis) are living in the Netherlands and this number has been rising annually [15]. These Indians are relatively homogenous in terms of shared beliefs and practices related to oral health. Hence, the aim of this study was to assess the association of integration with self-reported oral health, oral health behaviours, and oral healthcare utilization among Indian migrants living in the Netherlands.

## Materials and methods

### Study design

For this study, we followed a cross sectional survey design. We gathered data on the Indian migrants via survey questionnaires. The Medical Ethics Review Board of the Medical Centre of the VU University Amsterdam approved the project (reference number 2020.479).

### Study population and sampling

As inclusion criteria, the study population consisted of randomly selected Indian adult migrants, aged 18 years and above, born in India and having stayed in The Netherlands for at least five years. As most Indian migrants are concentrated in five major cities, namely: Amsterdam, including Amstelveen, Utrecht, Rotterdam, The Hague, and Eindhoven [15], we included Indians from there. There were no exclusion criteria.

Rijksdienst voor Identiteitsgegevens (RvIG) authorized the Central Bureau of Statistics, Netherlands (CBS) to draw a stratified random sample from the population registry of Dutch Municipalities (Basis Registratie Personen (BRP). This paper is a part of the larger PhD project, where data were collected on the oral health among Indian migrants and the host population for comparisons. Hence, the sample size was calculated based on the number of natural teeth. Furthermore, to explore differences in the number of natural teeth between groups, a power calculation based upon detecting a minimum difference of two natural teeth (effect size = 0.21) using the following parameters: 80% power, 5% level of significance and a standard deviation of 9.4 natural teeth in Dutch adults (host population) aged 25–75 [16] was done. Finally, after compensating for missing data and the concentration of Indian migrants in the five cities mentioned above, we got the final sample of 1500 Indian migrants and 1500 host population (300 x each city), giving us our total sample size to be 3000. The sampling was performed in January 2021, and out of requested 1500 contact details of Indian migrants, we received the postal addresses of approximately 1,378 Indians.

### Data collection and processing

Between February 2021 and April 2021, all potential Indian participants (n = 1,378) were contacted with an invitation for participation at their postal addresses. This included an introduction letter, a questionnaire with the consent form and a return envelope. The letter also included a link for online submission of the questionnaire for those opting for the digital method. We used Qualtrics Online Survey Software (version February 2021) for the online submission of the questionnaire, which included the provision for digital consent before opening the questionnaire page. A first reminder letter was sent after three weeks. Following this, we sent the second reminder after next two weeks that included the last reminder letter and another copy of the questionnaire. The paper version of the questionnaire that was sent to the postal addresses was in English whereas the online version was available in English, Hindi, and Dutch. The access to contact the participants was reserved with only the principal investigator, who assigned codes to all questionnaires, following which the data from the questionnaires was entered anonymously for analysis.

### Variables recorded

Oral health status was the first outcome variable, measured using the measure of self-rated oral health used in the GLOBE study, Netherlands: ‘How would you rate your oral health,’ with ordinal response categories grouped as: good (very good and good) versus bad (fair to very poor) (16). We also used the oral impact on daily performances (OIDP) scale, a pre-validated questionnaire, measured on a six-point Likert scale to reflect how severe the impact of each event was, ranging from 0 (indicating no impact), to 5 (indicating a very severe impact) [17]. Sum scores were created by adding the nine OIDP items as assessed originally. Finally, the OIDP frequency scores were also dichotomized, the responses as either no (OIDP score of 0) or yes (with OIDP score of 1 or higher score) [17]. Furthermore, we asked questions pertaining to oral disease burden as ‘Do your gums usually bleed?’ or ‘Do you think your teeth are loose (mobile) in your mouth?’ The responses for these were dichotomized as either yes or no.

The second outcome variable was oral health behaviours for which we included questions on habits, such as smoking and alcohol consumption [18], frequency of sugar consumption in the form of cakes and chocolates, fizzy drinks, and addition of sugar in hot beverages [18, 19] with the responses being never, monthly, or weekly. We also asked questions on ‘How do you clean your teeth?’ with manual toothbrush, electric toothbrush, or both as response options and ‘fluoride use in toothpaste,’ with dichotomized response as yes or no.

Oral healthcare utilization was the third outcome variable and was assessed with the questions on visiting the dental professional with the response category: no visits, visit only the dentist, visit only the dental hygienist, or visit both. In addition, we asked ‘How satisfied are you with the dental care provided to you in the Netherlands?’ with responses on a Likert scale: satisfied or neutral or unsatisfied [19, 20]).

The questionnaire was used to collect information on socio-demographic characteristics, notably age, gender, country of birth, marital status, education, occupation, income, and dental insurance. In addition, participants were asked about their migration status, including the year of coming to the Netherlands, duration of stay, reason for coming to the Netherlands, and having a permanent residence.

Integration was the independent variable and was assessed using the Immigration Policy Lab (IPL) scale. IPL measures six dimensions: economic, psychological, social, linguistic, navigational, and political, and can either have four questions per domain (IPL-24) or two questions per domain (IPL-12) scale [13]. This scale provides a comprehensive and multifaceted overview of the level of integration of migrants in the host country. This scale is one of the few migrant integration indices, which is thoroughly checked for construct validity using both ‘contrasted groups’ approach and correlation with well-established predictors of integration from the literature [13]. The use of the IPL-12 scale has been suggested for descriptive analysis and though a shorter version, it does capture the overall measures of integration. The questions asked were ‘How connected do you feel with The Netherlands,’ with responses on a five-point Likert scale scoring extremely close connection as five points to no connection at all as one point (Psychological). Or ‘In the last 12 months, how often did you eat dinner with Dutch who are not part of your family’, with responses ranging from almost every day as five points to never scored as one point (social). The IPL-12 scale is an aggregate scale ranging from scores 12–60, with higher scores indicative of higher integration. Furthermore, we also included the Everyday discrimination scale (EDS) as another independent variable with eight items, having questions such as ‘Based on your background, are you treated with less politeness’ and the responses dichotomized as yes or no [21].

### Data analysis

Data from questionnaires were processed for analysis using IBM SPSS Statistics for Windows, version 27.0 (IBM Corp., Armonk, N.Y., USA). We used descriptive statistics to report the frequency distribution of all independent and dependent variables. Further, for all categorical variables, we used the chi-square test of independence to compare proportional differences between different categories among the Indian migrants.

We used univariate regression analysis (logistic regression for binary outcomes, ordinal regression for rank and responses on a Likert scale, and multinomial regression for nominal outcomes with more than two categories) to explore and assess the within group differences for the three outcome variables. Thereafter, we used multivariable regression analyses to describe the association of integration with all three oral health outcomes. This was done while adjusting for the selected covariables such as age, gender, marital status, education level, occupation, and income. For each variable in the equation, the following statistics were calculated: estimated odds ratio (exp[B]) and confidence intervals (CI). A p-value of <0.05 was used as an arbitrary cut-point for statistical significance.

## Results

We received the postal addresses of 1,387 Indian migrants from the CBS. One hundred and forty-two envelopes with the invitation were returned as participants had moved houses and were no longer registered to those addresses. Overall, a total of 147 Indians out of the remaining 1,245 contacted Indian migrants responded, giving a response rate of 12%. We received only one incomplete questionnaire, which was excluded from the analysis. Table 1 shows the demographic composition of the Indian migrants along with univariate analysis of all demographic variables with the integration (IPL-12) scale and everyday discrimination scale (EDS). Univariate analysis showed that Indian migrants having a Dutch passport, planning to settle in The Netherlands, and having (applied for) a permanent residence had significantly high integration scores. Also, Indians who had stayed in the Netherlands for a long and had come here to study or work were significantly more integrated. Furthermore, Indians with paid jobs had significantly higher integration scores compared to the ones with low-paid jobs. For discrimination, the proportion of Indians who were married and those in the low-income category were more discriminated against compared to singles or high-income Indian migrants.

**Table 1:** Descriptive analysis of demographic characteristics of 147 Indian migrants living in the Netherlands, including univariate analysis with IPL-12 and everyday discrimination scale (EDS).

Demographic characteristics	Descriptive statistics	Univariate analysis				
		**IPL-12 scale with range (12–60)**		**Discrimination** **(Yes/ No)**		
	**n (%)**	**Mean (SD)**	**p value**	**Yes** **n (%)**	**No** **n (%)**	**p value**
**Gender**						
Males	95 (65)	40.21 (5.66)	0.82	18 (55)	77 (68)	0.10
Females	52 (35)	40.48 (7.78)		15 (46)	36 (32)	
**Marital status**						
Married/cohabiting	117 (80)	40.67 (6.13)	0.18	21 (64)	95 (84)	0.01
Single/ divorced	30 (20)	38.90 (7.60)		12 (36)	18 (16)	
**Nationality as in passport**						
Indian	93 (63)	38.13 (5.46)	<0.00	21 (64)	72 (64)	0.96
Dutch	54 (37)	44.06 (6.38)		12 (36)	41 (36)	
**Settle in Netherlands**						
Yes	95 (64)	42.15 (6.22)	<0.00	20 (61)	74 (65)	0.60
No	52 (35)	36.94 (5.54)		13 (39)	39 (35)	
^**†**^ **Permanent residence (PR) status**						
Yes	72 (49)	43.35 (6.15)	<0.00	17 (52)	54 (48)	0.40
Applied for	19 (13)	37.89 (3.87)		2 (6)	17 (15)	
No	56 (38)	37.21 (5.78)		14 (42)	42 (37)	
**Education level**						
Low to medium educated	23 (16)	38.61 (6.20)	0.17	4 (12)	19 (17)	0.51
Highly educated	124 (84)	40.62 (6.49)		29 (88)	94 (83)	
**Occupation**						
Paid job	128 (87)	40.90 (6.15)	0.00	26 (79)	101 (90)	0.11
Unemployed/unable	19 (13)	36.32 (7.30)		7 (21)	12 (10)	
**Income**						
Low income	19 (13)	40.11 (7.38)	0.07	9 (27)	10 (9)	0.00
Medium income	28 (19)	37.93 (6.60)		9 (27)	19 (17)	
High income	99 (68)	41.08 (6.14)		15 (46)	83 (74)	
**Dental insurance**						
Yes	76 (52)	40.33 (6.34)	0.96	16 (48)	59 (52)	0.70
No/ I do not know	71 (48)	40.28 (6.64)		17 (52)	54 (48)	
**Religion you follow**						
None	80 (54)	39.25 (6.19)	0.06	16 (49)	64 (57)	0.82
Hinduism	36 (25)	40.44 (6.30)		9 (27)	26 (23)	
Sikhism	13 (9)	43.92(6.80)		4 (12)	9 (8)	
Others	18 (12)	42.11 (6.95)		4 (12)	14 (12)	
^**†**^ **Reason to move to The Netherlands**						
Family, personal reasons	36 (25)	39.97 (7.19)	0.01	10 (30)	26 (23)	0.69
Work or study	102 (69)	39.90 (5.88)		21 (64)	80 (71)	
Security, other reasons	9 (6)	46.22 (7.66)		2 (6)	7 (6)	
	**Median** **[IQR]**	**Correlation coefficient**		**n (Mean rank)**		
°**Age** (In years)	36 [Q3 = 43, Q1 = 32]	r (147) = 0.35, p<0.00		33 (62.42)	113 (76.73)	0.00
°**Duration of stay in Netherlands** (In years)	8 [Q3 = 14, Q1 = 5]	r (147) = 0.55, p<0.00		33 (76.03)	113 (72.76)	0.69

° **Age, Duration of stay in Netherlands- not normally distributed.** Spearman correlation, Mann Whitney U

**†**One way ANOVA (post hoc)- Indians who had a permanent residence had statistically significantly higher mean score of integration compared to other two groups.

**†**One way ANOVA (post hoc)- Indians who came for work or study had statistically significantly higher mean score of integration compared to the other two groups

While assessing all three oral health outcomes with IPL-12 and EDS, we found no significant associations with discrimination scale in univariate analysis, hence for multiple regression analysis, we did not include this scale. Table 2 shows the multiple regression analysis of the IPL-12 scale with the first outcome: self-reported oral health. In an adjusted model, highly integrated Indians had lower odds of reporting their oral health as fair to poor than those with lower integration [OR = 0.92(95%CI:0.85;0.99)]. No significant associations were seen with other oral health status variables.

**Table 2:** Association of integration (IPL-12) with self-reported oral health of Indian migrants living in the Netherlands, using multiple regression analysis.

	Self-rated oral health		OIDP impact		Diagnosed with gum diseases		Bleeding gums in last 3 months		Toothache in the last 3 months		Loose teeth		^Ɨ^ OIDP sum score	
	*Very good to good (Ref)* *Fair to poor*		*No (Ref)* *Yes*		*No (Ref)* *Yes*		*No (Ref)* *Yes*		*No (Ref)* *Yes*		*No (Ref)* *Yes*			
	**OR** **(95% CI)**	**p-value**	**OR** **(95% CI)**	**p-value**	**OR** **(95% CI)**	**p-value**	**OR** **(95% CI)**	**p-value**	**OR** **(95% CI)**	**p-value**	**OR** **(95% CI)**	**p-value**	**Unstandardized coefficient B (95% CI)**	**p-value**
**Crude OR**	0.97 [0.92;1.02]	0.34	0.94 [0.89;1.00]	0.08	1.01 [0.94;1.07]	0.77	0.98 [0.92;1.08]	0.92	0.94 [0.87;1.00]	0.07	0.95 [0.87;1.04]	0.31	-0.43 [-0.74; 0.11]	0.00
Adjusted OR	0.92 [0.85;0.99]	0.04	0.98 [0.90;1.07]	0.71	0.96 [0.87;1.06]	0.45	1.01 [0.93;1.10]	0.69	0.92 [0.82;1.02]	0.12	0.86 [0.74;1.00]	0.06	-0.23 [-0.63, -0.17]	0.25

Adjusted for Age, Gender, education, occupation, income, settle in the Netherlands, duration of stay in the Netherlands.

ɨ OIDP (Oral impact on daily performances) sum score: Linear regression analysis.

For other outcomes, binary regression analysis was used.

Table 3 shows the multiple regression analysis of IPL-12 with oral health behaviours outcomes (Habits and OHP-oral hygiene practices). In the adjusted model, Indians with higher integration had lower odds of using a manual toothbrush as compared to electric toothbrush or use of both [OR = 0.86(95% CI:0.76;0.97)]. In Table 4, multiple regression analysis of IPL-12 with oral health behaviours outcomes (diet in the form of sugar intake) was observed. Highly integrated Indians had lower odds of consuming Indian sweets more frequently than those Indians with lower integration [OR = 0.91(95% CI:0.86;0.97)]. Table 5 shows the multiple regression analysis of IPL-12 with oral healthcare utilization. When observing place of dental visits, Indians with higher integration had higher odds of visiting a dental professional in the Netherlands compared to visiting in both places: India and the Netherlands [OR = 1.15(95%CI:1.03;1.29)] in a fully adjusted model. Also, highly integrated Indians had lower odds of being unsatisfied with the dental care provided in the Netherlands [OR = 0.82(95% CI:0.74;0.91)].

**Table 3:** Association of Integration (IPL-12) with oral health behaviours (Habits and OHP-oral hygiene practices) of Indian migrants living in the Netherlands, using multiple regression analysis.

	Smoking [BINARY LOGISTIC REGRESSION]		Chewing tobacco [BINARY LOGISTIC REGRESSION]		Alcohol consumption [ORDINAL LOGISTIC REGRESSION]		OHP-cleaning teeth [MULTINOMIAL LOGISTIC REGRESSION]				Fluoride use in toothpaste [BINARY LOGISTIC REGRESSION]		Alternate methods to clean mouth [BINARY LOGISTIC REGRESSION]	
	*Yes* *No (ref)*		*Yes* *No (ref)*		*Never* *Monthly* *Weekly(ref)*		*Manual toothbrush* *Electric toothbrush* *Use both (ref)*				*Yes (ref)* *No*		*Yes* *No(ref)*	
							*Manual VS Both*		*Electric Vs Both*					
	**OR** **[95% CI]**	**p value**	**OR** **[95% CI]**	**p value**	**OR** **[95% CI]**	**p value**	**OR** **[95% CI]**	**p value**	**OR** **[95% CI]**	**p value**	**OR** **[95% CI]**	**p value**	**OR** **[95% CI]**	**p value**
Crude OR	0.98 [0.90;1.07]	0.65	0.96 [0.91;1.01]	0.12	1.06 [1.00;1.12]	0.03	0.84 [0.76;0.92]	<0.00	0.97 [0.89;1.05]	0.47	0.93 [0.88;0.98]	0.00	0.99 [0.92;1.05]	0.69
Adjusted OR	0.99 [0.86;1.14]	0.87	1.00 [0.92;1.09]	0.97	1.08 [0.99;1.16]	0.07	0.86 [0.76;0.97]	0.01	0.94 [0.83;1.06]	0.30	0.99 [0.92;1.07]	0.80	0.92 [0.83;1.02]	0.12

Adjusted for age, gender, education, occupation, income, settle in the Netherlands, duration of stay in the Netherlands.

**Table 4:** Association of integration (IPL-12) with oral health behaviours (diet) of Indian migrants living in the Netherlands, using multiple regression analysis.

	Indian sweets (mithai) [ORDINAL LOGISTIC REGRESSION]		Cakes, chocolates [ORDINAL LOGISTIC REGRESSION]		Fizzy drinks [ORDINAL LOGISTIC REGRESSION]		Sugar in hot beverages [BINARY LOGISTIC REGRESSION]		Sugar consumption since moving to Netherlands [ORDINAL LOGISTIC REGRESSION]	
	*Never* *Monthly* *Weekly (ref)*		*Never* *Monthly* *Weekly (ref)*		*Never* *Monthly* *Weekly (ref)*		*Yes (ref)* *No*		*Increased* *Decreased* *Same (ref)*	
	**OR** **[95% CI]**	**p value**	**OR** **[95% CI]**	**p value**	**OR** **[95% CI]**	**p value**	**OR** **[95% CI]**	**p value**	**OR** **[95% CI]**	**p value**
**Crude OR**	0.93 [0.88;0.97]	0.00	0.96 [0.91;1.01]	0.12	1.00 [0.95;1.05]	0.89	1.08 [1.01;1.14]	0.01	1.03 [0.98;1.08]	0.30
**Adjusted OR**	0.91 [0.86;0.97]	0.00	0.97 [0.91;1.04]	0.45	1.02 [0.95;1.08]	0.64	1.06 [0.98;1.15]	0.12	1.00 [0.94;1.08]	0.81

Adjusted for age, gender, education, occupation, income, settle in the Netherlands, duration of stay in the Netherlands.

**Table 5:** Association of Integration (IPL-12) with oral health care utilization of Indian migrants living in the Netherlands, using multiple regression analysis.

	Place of dental professional visit [MULTINOMIAL REGRESSION ANALYSIS]						Satisfaction with dental professional in the Netherlands [ORDINAL LOGISTIC REGRESSION ANALYSIS]	
	*Both places (ref)* *India* *Netherlands* *No dental visits*						*Satisfied* *Neutral* *Unsatisfied (ref)*	
	No visit Vs Both		Netherlands Vs Both		India Vs Both			
	**OR** **[95% CI]**	**p value**	**OR** **[95% CI]**	**p value**	**OR** **[95% CI]**	**p value**	**OR** **[95% CI]**	**p value**
**Crude OR**	0.89 [0.80;0.99]	0.02	1.21 [1.10;1.32]	<0.00	0.91 [0.84;0.99]	0.04	0.85 [0.79;0.91]	<0.001
**Adjusted OR**	0.88 [0.77;1.01]	0.07	1.15 [1.03;1.29]	0.01	0.93 [0.84;1.03]	0.16	0.82 [0.74;0.91]	<0.001

Adjusted for age, gender, education, occupation, income, settle in the Netherlands, duration of stay in the Netherlands.

## Discussion

In our study, we found that highly integrated Indian migrants had better self-reported oral health, made use of electric toothbrushes more, and consumed Indian sweets less frequently than those with lower integration scores. For dental visits, highly integrated Indians preferred to see a Dutch dental professional and were satisfied with the Dutch dental care available compared to the Indian migrants with lower integration.

Although clinical parameters such as dental caries and periodontal diseases related to oral health have been discussed, there is a scarcity of studies highlighting self-reported oral health among migrants and its association to various determinants of migration and acculturation. In a systematic review, Dahlan et al. [22] found that migrants with higher social integration reported their oral health to be better than those with lower scores, which is similar to our findings.

Batra et al highlighted that Indians in the United States of America (USA), scored high on oral health attitude as cleaning of the mouth is not only a religious practice but rinsing of the mouth after meals is a social norm as well [23] In our study, we found that Indians who had higher integration scores made use of electric toothbrush more than manual toothbrush. Since the type of toothbrushes used by migrants is not reported in most studies, it was not possible to make interpretations on this finding. Having said that, in their two systematic reviews, Dahlan et al. [10, 22]. did observe that host language proficiency, one of the proxies of acculturation was associated with better oral health practices among migrants. This could be attributed to differences in measuring oral hygiene practices and hence more studies are needed in the context of integration and its role in oral health behaviours among the migrant population.

Cultural influences are highly reflective in the food habits of people across the globe and for most Indians, sweets (Indian sweets called as Mithai) are integral to Indian traditions, especially the Indian festivals. Dahlan et al. [10]. also mention how country of birth can be an indicator of acculturation and can reflect the background on oral health beliefs and customs attached to migrants. In our study, we observed that highly integrated Indians consumed Indian sweets less frequently compared to the ones with lower integration. Literature reports that consumption of sugar in the form of sweets, cakes, and chocolates is lower among South Asians, including Indian migrants, compared to the host population although it is not clear what was included under the sweets category [23]. Hence, the findings in our study could be the influence of integration where interactions with host society resulted in dietary shift.

Indians with higher integration visited a Dutch dental professional rather than visiting in both, India as well as in the Netherlands. Also, highly integrated Indians were satisfied with the dental care provided in the Netherlands. Even though there is no published literature on the preference of dental visits in the home country versus the host country among the Indian migrants, Al-Haboubi et al. [24]. also reported that Asians, including Indians, had greater dental care utilization compared to other ethnic groups. In addition, Arora et al. [25]. reported the barriers in oral healthcare utilization, especially among the Indian migrants, as more culturally related since routine dental visits are not a norm in Indian society. Proxy measures such as host language proficiency, duration of stay in the host country, and age at migration have all been shown to have considerable influence on positive utilization of oral healthcare among migrants [10, 23]. However, more research, using better research designs, focusing on the role of integration in oral healthcare utilization is needed.

### Strengths and limitations

The findings from our study should be viewed as a starting point for assessing the association of integration with existing self-reported oral health among Indian migrants. Firstly, a unique feature of our study is that we included only Indian migrants from the Indian subcontinent. We thereby addressed the limitation of heterogeneity, as mentioned in numerous studies, on the oral health of migrants and ensured that the background characteristics were as homogenous as possible on social and cultural aspects. Secondly, we used a validated scale of integration: the IPL-12 scale to measure oral health among the migrants. Dental literature commonly uses global proxy measures of acculturation when reporting oral health among migrants. By using the IPL-12 scale, we addressed the lack of empirical measures of integration among migrants in other studies. Lastly, we incorporated variables closely related to cultural habits and oral health behaviours of Indians such as consumption of Indian sweets (Mithai) the place of dental visits (India or the Netherlands), and the type of toothbrush use in the Netherlands. The inclusion of culturally specific variables may be useful in assessing the role of integration on oral health among specific migrant groups.

However our study presents certain limitations as well. The response rate in our study was low (11%). Although we used random sampling method to send out our survey questionnaire, we had access only to the postal addresses of Indians. It has been documented that migrants are usually a hard-to-reach group and to increase their participation, one needs to resort to community involvement and include the possibility of direct contact with the migrants. As this study was undertaken during the COVID lockdown period, it was not feasible to contact Indians in any other way other than sending reminders to increase response rates. Secondly, the cross-sectional study design does not allow for any causal inference. Furthermore, normative measures are valuable in diagnosing and treatment of oral diseases, but perceived measures help in assessing the individual’s oral health subjectively, taking into account their experiences, beliefs, and satisfaction level. However, the use of questionnaires for self-reported oral health status may have led to subjective and recall bias [26]. Hence the results of our findings need to be interpreted with caution.

## Conclusion

In conclusion, our results show that integration has a clear association with self-reported oral health, behaviours, and oral healthcare utilization among Indian migrants. That is, highly integrated Indians perceive better oral health, and show more beneficial oral health behaviours, while they visit a Dutch dental professional and are more satisfied with the oral healthcare in the Netherlands. This calls for dental practitioners in the Netherlands to provide a supportive environment for Indian migrants, and encourage them to adopt beneficial oral health behaviours, while motivating them to make appointments for dental checkups. By being mindful of the benefits of integrating the Indian migrant population at both, cultural and social levels, policy makers can contribute towards achieving equal opportunities for oral healthcare utilization for all. Further, to achieve comprehensive and comparable data on oral health among migrants, research designs including multidimensional scales of acculturation in longitudinal studies or qualitative research methods will help refine and better our understanding of the role of integration on oral health. In addition, the use of clinical parameters rather than solely relying on self-reported data can enhance the validity of research further.

## Supporting information

S1 File (PDF)
